# SP70 is a novel biomarker of hepatocellular carcinoma

**DOI:** 10.3389/fonc.2023.1149397

**Published:** 2023-04-06

**Authors:** Lin Wang, Hui Shi, Jia Wei, Wen-Xiu Chen, Yue-Xinzi Jin, Chun-Rong Gu, Yuan Mu, Jian Xu, Shi-Yang Pan

**Affiliations:** ^1^ Department of Laboratory Medicine, the First Affiliated Hospital of Nanjing Medical University, Nanjing, China; ^2^ Branch of National Clinical Research Center for Laboratory Medicine, Nanjing, China; ^3^ NHC Contraceptives Adverse Reaction Surveillance Center, Jiangsu Health Development Research Center, Nanjing, China

**Keywords:** hepatocellular carcinoma, SP70, early recurrence, diagnostic performance, recurrence-free survival

## Abstract

**Background:**

Tumor-specific protein 70 (SP70) was identified as a new biomarker associated with the proliferation and invasion of cancer cells. This study aimed to investigate the expression of SP70 in hepatocellular carcinoma (HCC) and assess its clinical value in the diagnosis and prediction of early HCC recurrence.

**Methods:**

A total of 1049 subjects from the First Affiliated Hospital of Nanjing Medical University were recruited in this study. Serum SP70, alpha-fetoprotein (AFP) and prothrombin induced by vitamin K absence II (PIVKA-II) were measured. The diagnostic performance for HCC was obtained using the receiver operating characteristic (ROC) curve, and recurrence-free survival (RFS) was calculated using the Kaplan–Meier method. Univariate and multivariate analyses were performed to identify predictive factors of RFS.

**Results:**

SP70 was highly expressed in HCC cells and HCC tissue. Serum SP70 levels in the HCC group were significantly higher than in the benign liver diseases group and healthy control group (*P*<0.001). SP70 combined with AFP showed the best diagnostic performance (AUC=0.909, 95%CI [confidence interval]=0.890–0.929). Kaplan–Meier analysis revealed that patients with high SP70 levels had shorter median RFS than those with low SP70 levels (*P*=0.003). In addition, high SP70 levels were significantly associated with shorter RFS (*P*=0.037) in the AFP-negative subgroup. Univariate and multivariate analyses confirmed that preoperative serum SP70 level, serum AFP, tumor diameter and microvascular invasion were independent prognostic factors of RFS.

**Conclusion:**

SP70 is a promising biomarker in diagnosing HCC. High preoperative serum SP70 level is associated with an increased risk of early relapse and could be used as a valuable marker to predict early recurrence of HCC after resection.

## Introduction

Liver cancer represents the sixth most common malignancy worldwide and the fourth leading cause of cancer-related death (1, 2). Hepatocellular carcinoma (HCC) accounts for 90% of all new liver cancer cases, and approximately half of cases are attributed to hepatitis B virus (HBV) infection. HCC constitutes a serious health problem, especially in China, where HBV is the most predominant risk factor. Currently, the diagnosis of HCC mainly depends on biopsy and imaging techniques, but a large portion of patients are diagnosed at an advanced stage. HCC has a dismal prognosis, with a 5-year overall survival rate of less than 15%, because of the high incidence of metastasis and recurrence after surgery and limited therapeutic options. Therefore, it is necessary to identify novel sensitive biomarkers for early diagnosis and prediction of HCC recurrence. Recent large-scale genomic studies have been performed to explore multiple mutations for diagnostic biomarkers and potential therapeutic targets (3–5), but there is still a long way to translate these findings into clinical practice.

Alpha-fetoprotein (AFP) has been the most utilized surveillance biomarker for HCC worldwide. However, its role has been controversial for decades due to the lack of sensitivity (6). In addition, prothrombin induced by vitamin K absence II (PIVKA-II), considered another biomarker of HCC unrelated to AFP, has been reported to exhibit a better diagnostic performance than AFP (7, 8). Nevertheless, the clinical utility of PIVKA-II remains uncertain due to substantial variations in its diagnostic efficiency across most studies and the lack of outcome prediction (9, 10).

We previously reported a new antigen with the relative molecular mass (Mr) of 70 kDa—tumor-specific protein 70 (TSP70 or SP70)—recognized by the monoclonal antibody (McAb) named NJ001. As a tumor derived antigen, SP70 is widely involved in the regulating the expression of numerous genes (GEO accession number: GSE59655), promoting cancer cell proliferation and metastasis. Serum SP70 levels were significantly correlated with tumor differentiation and lymph node metastasis and played an important role in cancer chemotherapy monitoring (11, 12). Moreover, SP70-targeted imaging was found to be able to markedly improve the detection of lung adenocarcinoma detection (13). Recent results showed that SP70 was also expressed in HCC. However, the significance of SP70 detection in HCC remains unclear. The aim of this study was to explore the clinical value of SP70 in the diagnosis and prediction of early HCC recurrence.

## Material and methods

### Study population

A total of 1049 subjects from the First Affiliated Hospital of Nanjing Medical University were enrolled in this study between October 2017 and March 2019, including 365 HCC patients, 310 patients with benign liver diseases (BLD) and 374 healthy volunteers. The BLD group included 101 patients with liver cirrhosis (LC), 100 with chronic hepatitis, 53 with hepatic hemangioma, 35 with hepatic cyst, 18 with hepatapostema and 3 with focal nodular hyperplasia. Among the HCC patients, 168 patients who underwent hepatectomy were followed up. Patients with HCCs were confirmed by pathology, and who had complete clinical data were included in the study. We excluded patients: 1) with other malignant tumors; 2) recurrent HCC; 3) who received preoperative anti-cancer treatment; and 4) who died during the perioperative period. All patients with benign liver diseases were followed up for 6 months after sampling to confirm non-HCC. The staging was assessed using the Barcelona Clinic Liver Cancer (BCLC) staging system.

### Measurements of serum tumor markers

All blood samples were centrifuged at 3000 rpm for 10 minutes at room temperature for serum separation within 2 hours after collection. Serum SP70 was detected using an enzyme-linked immunosorbent assay (ELISA) kit (Code Biotech, Jiangsu, China). Serum AFP and PIVKA-II levels were measured on a Roche cobas e602 automated analyzer (Roche, Mannheim, Germany) and LUMIPULSE G1200 immunochemistry system (FujirebioInc, Tokyo, Japan), respectively.

### Immunohistochemistry

To determine the expression of SP70 in liver tissues, we collected HCC and corresponding adjacent tissues. Paraffin-embedded samples were sectioned at 4 mm thickness, and SP70 expression was detected with immunohistochemistry (IHC) kit (Code Biotech). All the specimens were independently examined by two investigators.

### Cell culture

Human HCC lines Hep3B, Huh7 and normal human hepatic cell L02 were purchased from the cell bank of the Chinese Academy of Sciences in Shanghai. DMEM (Gibco, Grand Island, NY, USA) supplemented with 10% FBS (Gibco) was used as the culture medium for all HCC cell lines. The RPMI-1640 medium (Gibco) supplemented with 10% FBS (Gibco) was used for L02 cell culture. Cell lines were incubated in a humidified atmosphere containing 5% CO_2_ at 37°C.

### Immunofluorescence analysis

Direct immunofluorescence was performed as follows. A dose of 100 μg NJ001 was labeled with Mix-n-StainTM CF^®^ 488A Antibody Labeling Kit (Biotium, California, USA) for fluorescence imaging analysis and the final concentration of NJ001-488A was 250 μg/mL. Briefly, cells were grown to 80% confluence on coverslips and then fixed in 95% ethanol at room temperature. After washing with PBS, the cell membranes were broken in 0.25% Triton X-100 for 15 min, blocked with 3% BSA for 2 hours at room temperature, and then incubated with diluent (1:50) NJ001-488A for 2 h at 37°C. Finally, the slides were washed with PBS three times and sealed with Antifade Mounting Medium with DAPI (Beyotime Biotechnology) and visualized using a Nikon Eclipse Ti fluorescence microscope (Nikon, Melville, NY, USA).

### Follow-up for recurrence

All HCC patients who underwent curative resection were followed up. Liver ultrasonography and tumor marker assays were performed every 1–2 months after surgery. Suspected tumor recurrence in the liver was confirmed using enhanced computed tomography (CT) or magnetic resonance imaging (MRI) or biopsy. Recurrence-free survival (RFS) was defined as the time from curative surgery to recurrence.

### Statistical analysis

All statistical analyses were performed with SPSS for Windows, version 22 (SPSS Inc., Chicago, IL, USA). Data were expressed as median and interquartile range. Differences between categorical variables were assessed using the Pearson’s chi-squared test or Fisher exact test. Mann–Whitney and Kruskal–Wallis tests were used to assess differences between groups of nonparametrically distributed continuous variables. Bivariate correlations were evaluated using Spearman rank correlation coefficient. A *P*-value of <0.05 was considered significant. Diagnosis-related indicators obtained from receiver operating characteristics (ROC) analysis, including sensitivity, specificity and the area under the curve (AUC), were used to assess the performance of tumor markers. The Cox proportional hazards regression model was performed to identify the independent predictive factors of RFS. A cut-off of *P*<0.05 was used to select variables entered into the multivariate model from the univariate analysis. Kaplan-Meier analysis with the log-rank test was utilized to compare RFS of different risk groups, and *P*<0.05 was considered statistically significant.

## Results

### The expression of SP70 in liver cell lines and tissues

**Figure 1:**
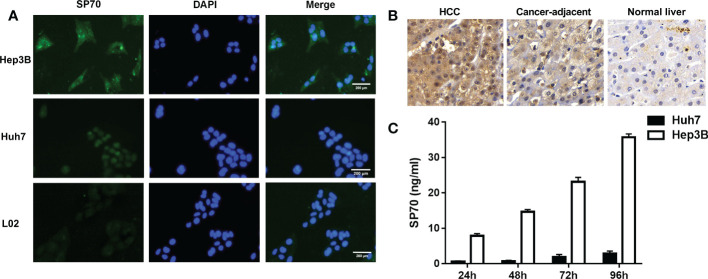
The expression of SP70 in HCC cells and tissues. **(A)** Immunofluorescence analysis showed that SP70 was highly expressed in Hep3B cells, mainly expressed in the nucleus of Huh7 cells and lowly expressed in L02 cells; **(B)** Immunohistochemistry staining showed that SP70 was highly expressed in HCC tissues (×400); **(C)** Levels of SP70 in culture supernatant increased further with the proliferation of HCC cells.

The results of indirect immunofluorescence showed that SP70 was highly expressed in Hep3B cells, mainly expressed in the nucleus of Huh7 cells and lowly expressed in L02 cells (**Figure 1A**). We detected SP70 in cell culture supernatant by ELISA and found that the levels of SP70 increased with the proliferation of HCC cells (**Figure 1C**). Immunohistochemistry analysis indicated that the expression of SP70 in HCC was higher than that in cancer-adjacent tissues and normal liver tissue (**Figure 1B**). Furthermore, Spearman correlation analysis showed that SP70 expression in HCC tissues and serum SP70 were positively correlated (r=0.402, *P*<0.05).

### Patient characteristics

**Figure 2:**
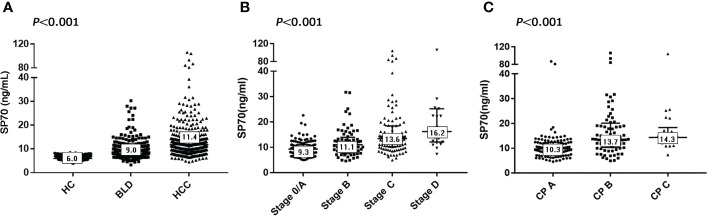
SP70 levels in different groups and associations with BCLC and CP stages. **(A)** Serum levels of SP70 in the HCC, BLD and HC groups; **(B)** SP70 associations with BCLC stage; **(C)** SP70 associations with CP stage. The median level of SP70 for each group has been marked in the appropriate box.

To evaluate the potential application of SP70 in HCC, we divided the 1049 study participants into three groups. The HCC group had significantly higher SP70 levels compared with the BLD group and healthy controls (HC) (all *P*<0.001). The median levels of SP70 were 11.4 ng/mL, 9.0 ng/mL and 6.0 ng/mL in the HCC, BLD and HC groups, respectively (**Figure 2A**). In addition, SP70 levels were significantly higher in the BLD group compared with the HC group (*P*<0.001, respectively).

The characteristics of HCC patients and preoperative SP70 levels in subgroups are summarized in **Table 1**. Among the 365 HCC patients enrolled, 302 (82.7%) were men, and the median age was 57 years (range: 20–86). There were 4.8 times more men than women (17.3%). Chronic hepatitis B virus (HBV) infection was by far the most common underlying cause of liver diseases (n=299, 81.9%), far more than chronic hepatitis C virus (HCV) infection (n=14, 3.8%). Other factors included cryptogenic/other cirrhosis (n=13, 3.6%) and no underlying chronic liver disease (n=39, 10.7%). In the HCC group, 133 patients were diagnosed at BCLC stage 0/A (36.4%), 72 patients at stage B (19.7%), 137 patients at stage C (37.5%) and 23 patients at stage D (6.3%). Two-thirds of the patients could not be diagnosed early, causing about half of the patients to miss opportunities for radical resection and be prescribed other treatment options, including transarterial chemoembolization (n=120, 32.9%), ablation (n=24, 6.6%) and supportive care (n=49, 13.4%). Serum SP70 levels were significantly different in HCC patients grouped by tumor number, tumor diameter, BCLC stage, vascular invasion and intra- or extrahepatic metastasis (**Table 1**). Moreover, further analysis indicated that serum SP70 increased with advancing BCLC and Child-Pugh (CP) stage (**Figures 2B, C**).

**Table 1:** Clinicopathological characteristics and serum SP70 levels of HCC patients.

Clinical characteristic	n (%)	SP70 (ng/mL)	*P*
Sex			
Male	302 (82.7%)	10.5 (7.9, 13.7)	0.298
Female	63 (17.3%)	11.7 (9.2, 13.4)	
Age (years)			
≤57	187 (51.2%)	10.9 (8.3, 13.4)	0.790
>57	178 (48.8%)	10.1 (8.1, 13.6)	
Tumor diameter (cm)			
≤5	130 (35.6%)	10.3 (8.2, 13.5)	*0.019
>5	235 (64.4%)	12.0 (8.9, 15.8)	
Tumor number			
Single	259 (71.0%)	10.5 (8.3, 13.5)	*<0.001
Multiple	106 (29.0%)	13.7 (10.1, 20.3)	
Grade			
I-II	103 (50.0%)	9.7 (7.3, 12.2)	0.471
III	103 (50.0%)	9.5 (7.6, 12.4)	
Missing	159		
BCLC Stage			
Stage (0/A)	133 (36.4%)	9.4 (7.1, 11.9)	**<0.001
Stage (B/C/D)	232 (63.6%)	13.1 (9.7, 15.6)	
Vascular invasion			
Yes	225 (59.7%)	13.1 (9.6,18.0)	**<0.001
No	140 (40.3%)	9.7 (7.8, 12.3)	
Intra- or extrahepatic metastasis			
Yes	191 (52.3%)	13.4 (10.0, 18.7)	**<0.001
No	174 (47.7%)	9.8 (8.0, 12.4)	
HBV/HCV infection			
Yes	316 (86.6%)	11.8 (8.5, 14.8)	0.432
No	49 (13.4%)	10.2 (8.5, 13.6)	

BCLC, Barcelona clinic liver cancer; HBV, hepatitis B virus; HCV, hepatitis C virus.

*P<0.05, **P<0.001.

### The diagnostic value of SP70 in HCC

**Figure 3:**
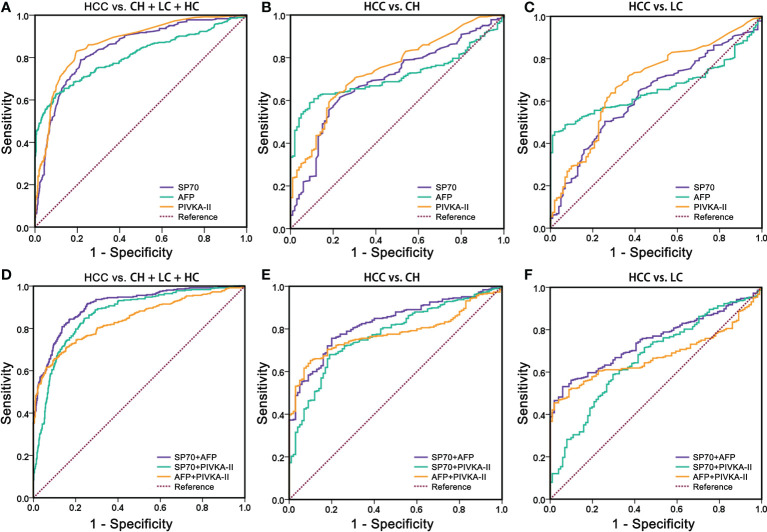
Comparison of receiver operating characteristics curves of SP70, AFP, PIVKA-II and three different multi-marker combinations. **(A, D)** HCC vs. BLD + HC; **(B, E)** HCC vs. BLD; **(C, F)** HCC vs. LC. BLD, benign liver diseases; HC, healthy control; LC, liver cirrhosis.

To evaluate the diagnostic performance of SP70, AFP and PIVKA-II in HCC, we performed ROC analysis (**Figure 3**). The findings revealed that SP70 could serve as a valuable marker for detecting HCC with a sensitivity of 70.0% and a specificity of 83.5% at a cut-off value of 9.3 ng/mL (AUC=0.840, 95% CI [confidence interval]=0.814–0.867). PIVKA-II (AUC=0.868, 95% CI 0.844–0.891) had a higher AUC value than AFP (AUC=0.807, 95% CI=0.776-0.838, *P*<0.001), and there was no significant difference between SP70 and PIVKA-II (*P*>0.05). **Figure 3B** shows the performance of SP70, AFP and PIVKA-II to distinguish between HCC from CH. The AUC values of SP70, AFP and PIVKA-II were 0.696 (95% CI=0.640–0.751), 0.715 (95% CI=0.670–0.760) and 0.747 (95% CI=0.696–0.798), respectively. However, there was no statistical difference among the AUC values of three markers (*P*>0.05). When distinguishing HCC from LC, the diagnostic performance of three markers decreased further. The AUC values were 0.627 (95% CI=0.569–0.685), 0.658 (95% CI=0.611–0.706) and 0.689 (95% CI=0.631–0.746) for SP70, AFP and PIVKA-II, respectively (**Table 2**). Similarly, no significant difference was observed among the AUC values of three markers (*P*>0.05).

**Table 2:** Diagnostic performance of SP70, AFP, PIVKA-II and marker combinations in detecting HCC.

	AUC	95%CI	Cut-off value	Sensitivity (%)	Specificity (%)
HCC vs. CH + LC + HC					
SP70 (ng/mL)	0.840	0.814–0.867	9.3	70.0	83.5
AFP (ng/mL)	0.807	0.776–0.838	27.97	62.7	90.1
PIVKA-II (mAU/mL)	0.868	0.844–0.891	30	83.0	80.3
SP70+AFP	0.909	0.890–0.929		84.1	83.1
SP70+PIVKA-II	0.867	0.843–0.891		84.7	76.0
AFP+PIVKA-II	0.841	0.814–0.869		65.8	90.1
HCC vs. CH					
SP70 (ng/mL)	0.696	0.640–0.751	10.0	61.4	77.0
AFP (ng/mL)	0.715	0.670–0.760	36.41	58.9	91.0
PIVKA-II (mAU/mL)	0.747	0.696–0.798	65	60.5	81.0
SP70+AFP	0.827	0.787–0.866		75.6	80.0
SP70+PIVKA-II	0.773	0.726–0.820		67.9	81.0
AFP+PIVKA-II	0.778	0.737–0.820		65.2	90.0
HCC vs. LC					
SP70 (ng/mL)	0.627	0.569–0.685	11.4	50.4	74.3
AFP (ng/mL)	0.658	0.611–0.706	253.58	45.5	98.0
PIVKA-II (mAU/mL)	0.689	0.631–0.746	56	63.8	71.3
SP70+AFP	0.748	0.704–0.792		53.2	94.1
SP70+PIVKA-II	0.666	0.609–0.723		58.9	70.3
AFP+PIVKA-II	0.676	0.629–0.723		51.8	91.1

HCC, hepatocellular carcinoma; CH, chronic hepatitis; HC, healthy control; LC, liver cirrhosis; AFP, alpha-fetoprotein; PIVKA-II, prothrombin induced by vitamin K absence II; AUC, areas under the curve.

We combined different markers to enhance their diagnostic performance. Comparisons of different two-marker combinations are shown in **Table 2**. Overall, the combination of SP70 and AFP showed the best diagnostic performance, which had a significantly better AUC (AUC=0.909, 95% CI=0.890–0.929) compared to a combination of SP70 and PIVKA-II (AUC=0.867, 95% CI=0.843–0.891, *P*<0.001), combination of AFP and PIVKA-II (AUC=0.841, 95% CI=0.814–0.869, *P*<0.001) and PIVKA-II (*P*=0.002) alone. When distinguishing HCC from CH, an AUC value of 0.827 (95% CI=0.787–0.866) could be obtained by combining SP70 and AFP, which was significantly higher than a combination of AFP and PIVKA-II (AUC=0.778, 95% CI=0.737–0.820, *P*=0.015), combination of SP70 and PIVKA-II (AUC=0.773, 95% CI=0.726–0.820, *P*=0.002) and PIVKA-II (*P*=0.009) alone. Likewise, the combination of SP70 and AFP showed a better diagnostic value in differentiating HCC from LC (AUC=0.748, 95% CI=0.704–0.792) than the other two combinations (*P*<0.001).

### SP70 as a predictor for early recurrence after surgery

**Figure 4:**
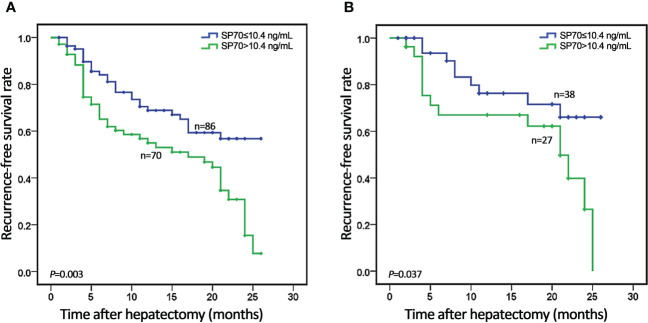
Kaplan–Meier analysis of recurrence-free survival (RFS) stratified by serum SP70 level. **(A)** Association of SP70 level with RFS in all 156 HCC patients; **(B)** Association of SP70 level with RFS in the AFP-negative subgroup.

A total of 168 cases were followed up after hepatectomy. None of the patients received postoperative adjuvant treatment. Twelve were lost to follow-up, giving a follow-up rate was 92.9%. During a median of 11 months of follow-up, 71 patients (45.5%) cases experienced HCC recurrence, including 64 (90.1%) with intrahepatic recurrence and 7 (9.2%) with extrahepatic metastasis. The 1- and 2-year recurrence-free survival rates were 62.3% and 33.2%, respectively. According to ROC analysis, the cut-off values of preoperative serum SP70, AFP and PIVKA-II for identifying early recurrence were 10.4 ng/mL, 20.94 ng/mL and 406 mAU/mL respectively. The clinical and biological features of HCC patients who underwent resection are available in **Table 3**. Kaplan–Meier analysis revealed that patients with high SP70 levels displayed worse median recurrence-free survival than those with low SP70 levels (*P*=0.003), **Figure 4A**. Furthermore, preoperative AFP was negative in 41.7% (n=65) of all patients followed up. Moreover, among the 71 patients with recurrence, 23 (32.4%) had a negative preoperative AFP. High SP70 levels were associated with significantly worse RFS in the AFP-negative subgroup (*P*=0.037), **Figure 4B**.

**Table 3:** Clinical and biological features of HCC patients undergoing resection according to the preoperative serum level of SP70.

Clinical characteristic	Low SP70 (≤10.4 ng/mL)	High SP70 (>10.4 ng/mL)	*P*
Sex			
Male	74	55	0.220
Female	12	15	
Age (years)			
≤57	38	37	0.281
>57	48	33	
Tumor diameter (cm)			
≤5	50	38	0.629
>5	36	32	
Tumor number			
Single	70	62	0.271
Multiple	16	8	
Grade			
I-II	43	32	0.594
III	43	38	
Ascites			
No	69	60	0.368
Yes	17	10	
Microvascular invasion			
No	56	41	0.402
Yes	30	29	
Cirrhosis			
No	29	23	0.909
Yes	57	47	
HBV/HCV infection			
No	19	9	0.135
Yes	67	61	
AFP			
≤20 ng/mL	38	27	0.479
>20 ng/mL	48	43	
PIVKA-II			
≤40 mAU/mL	25	29	0.107
>40 mAU/mL	61	41	

HBV, hepatitis B virus; HCV, hepatitis C virus; AFP, alpha-fetoprotein; PIVKA-II, prothrombin induced by vitamin K absence II.

The results of the univariate and multivariate analysis for RFS are shown in **Table 4**. In univariate analysis, RFS was significantly associated with tumor diameter (HR [hazard ratio]=2.512, 95% CI=1.562–4.040, *P*<0.001), tumor number (HR=1.863, 95% CI=1.033–3.361, *P*=0.039), microvascular invasion (HR=2.477, 95% CI=1.551–3.956, *P*<0.001), serum AFP (HR=2.115, 95% CI=1.269–3.526, *P*=0.004) and serum SP70 (HR=2.008, 95% CI=1.247–3.233, *P*=0.004). Multivariate analysis demonstrated that tumor diameter >5 cm (HR=1.952, 95% CI=1.182–3.222, *P*=0.009), microvascular invasion (HR=1.787, 95% CI=1.085–2.944, *P*=0.023), preoperative high-level SP70 (HR=1.927, 95% CI=1.177–3.156, *P*=0.009) and high-level AFP (HR=1.766, 95% CI=1.043–2.991, *P*=0.034) were independent prognostic factors of RFS.

**Table 4:** Univariate and multivariate analysis of risk factors predicting recurrence-free survival after radical hepatectomy in HCC patients.

Variable	Univariate analysis		Multivariate Analysis	
	HR (95%CI)	*P*	HR (95%CI)	*P*
Sex (female)	0.895(0.479–1.673)	0.728		
Age (>57 years)	0.947(0.594–1.511)	0.820		
Tumor diameter (>5 cm)	2.512(1.562–4.040)	**<0.001	1.952(1.182–3.222)	*0.009
Tumor number (multiple)	1.863(1.033–3.361)	*0.039	1.570(0.846–2.916)	0.153
Grade (III)	1.386(0.862–2.229)	0.178		
Microvascular invasion	2.477(1.551–3.956)	**<0.001	1.787(1.085–2.944)	*0.023
HBV/HCV infection	1.679(0.881–3.199)	0.115		
Cirrhosis	1.350(0.818–2.229)	0.240		
Ascites	1.552(0.859–2.803)	0.145		
AFP (>20.94 ng/mL)	2.115(1.269–3.526)	*0.004	1.766(1.043–2.991)	*0.034
PIVKA-II (>406 mAU/mL)	1.468(0.902–2.388)	0.122		
SP70 (>10.4 ng/mL)	2.008(1.247–3.233)	*0.004	1.927(1.177–3.156)	*0.009

HBV, hepatitis B virus; HCV, hepatitis C virus; AFP, alpha-fetoprotein; PIVKA-II, prothrombin induced by vitamin K absence II.

*P<0.05, **P<0.001.

## Discussion

Despite being one of the most common malignancies with poor survival globally, HCC is limited in diagnosis and treatment due to its atypical early symptoms and high recurrence rate. Although guidelines from the American Association for the Study of Liver Diseases (AASLD) and the European Association for the Study of the Liver (EASL) recommend surveillance to be primarily imaging-based (14, 15), it is usually difficult to distinguish malignant from benign hepatic lesions when the size of the lesion is small (less than 2 cm) (16). Pahwa et al. (17) reported that roughly 20% of presumed HCC nodules were inaccurately characterized, and many small HCC nodules remained undiagnosed using established imaging criteria. In addition, pathological biopsy, the golden standard for diagnosis of HCC, is limited because it is traumatic and accurate localization of small lesions and tumor heterogeneity is challenging. A negative biopsy result does not rule out HCC (18). Given these considerations, biomarkers are still important in improving HCC detection as their assessments are convenient, cost-effective, available and repeatable.

In this study, we first reported the role of SP70 in the diagnosis and prognosis of HCC. As a new potential biomarker, SP70 has been proved to be valuable in lung cancer and gastric cancer detection in previous studies (11, 19), while its expression and clinical value in HCC have not been studied. Our results showed the SP70 protein was mainly localized on the membrane and in the cytoplasm of HCC cells, and its expression in HCC tissues was stronger than in adjacent tissues and normal liver tissues. The HCC group had higher serum SP70 levels than the BLD and HC groups. Moreover, serum SP70 levels tended to increase with higher BCLC and CP stages, indicating SP70 may be vital for driving progression in HCC. Hepatocarcinogenesis is a slow, multistep process evolving from hepatitis, cirrhosis, and dysplasia to HCC. About 80% of all HCCs have a cirrhosis background (20). HBV drives the hepatocyte transformation, HCC development and progression through direct or indirect mechanisms, including chronic inflammation, DNA damage, chromosomal instability, epigenetic modification and early neovascularization (21). Of the 81 patients who provided tissues for immunohistochemistry, 76 (93.8%) had backgrounds of HBV-related cirrhosis accompanied by different levels of SP70 expression in adjacent samples. We speculated that the expression of SP70 reflected the malignant potential of cells. However, the clinical contribution of SP70 in differentiating benign and premalignant lesions needs further study.

For the diagnosis of HCC, AFP has high specificity but insufficient sensitivity. When using a cut-off level of 20 ng/mL, its sensitivities and specificities were 41%–65% and 80%–94%, respectively (22). AFP needs to be combined with imaging or other tumor markers for HCC screening. A large multicenter phase II study showed that the sensitivity of PIVKA-II in the diagnosis of early HCC was only 56% (9), and another case-control study demonstrated that PIVKA-II was more efficient than AFP with a sensitivity of 77% (10). Some researchers have advised combining AFP and PIVKA-II to improve diagnostic accuracy (23, 24). Lens culinaris agglutinin-reactive fraction of AFP (AFP-L3) is another recommended indicator for screening of HCC (25, 26). Unfortunately, AFP-L3 detection is not able to be provided in our lab. In the past decade, some circulating markers for diagnosing HCC have been reported, such as micro-RNA and circulating cell-free DNA in plasma, showing good diagnostic performance (27, 28). However, most detection methods require a long response time or high cost and have not been systematized and standardized, limiting their application in practice.

Considering the limited accuracy of single indicator, it is often necessary to combine other markers to improve the diagnostic performance. In this study, combining SP70 with AFP or PIVKA-II significantly improved the performance compared with using SP70 alone. This may be attributed to the special expression mechanism of SP70 unrelated to the other two indicators. However, when used for detecting early HCC, the combinations did not display satisfactory results as expected. Additionally, the heterogeneity of assay methods in evaluating the performance of markers should not be ignored. Serum AFP was detected by electrochemiluminescence (ECL), and PIVKA-II was measured by chemiluminescence (CL). Due to their advantages of having a wide linear range, high sensitivity, rapid analysis with a low background and high automatization, ECL and CL have been broadly applied in biomolecule detection (29). In contrast, the ELISA method for serum SP70 detection had obvious defects with a lower linear range, sensitivity and efficiency. The improved analytical sensitivity could improve the detection of lower levels of SP70 and make HCC early diagnosis practical after updating the assay into ECL or CL.

The poor survival of HCC is mainly attributed to the high risk of recurrence. Close to 70% of patients relapse after surgery (30). Two types of recurrence have mostly been described in the literature: 1) early recurrence, caused by metastasis from the original tumor (≤2 years); and 2) late recurrence, due to *de novo* tumors arising in the cirrhotic liver (>2 years) (31, 32). The mechanism of recurrence has not been completely elucidated. Molecular characterization of HCC using genomics, transcriptomics and proteomics has developed our understanding of the mechanism recurrence, but most findings come from retrospective studies without validation (33). In this study, serum SP70 level, serum AFP level, tumor diameter and microvascular invasion (MVI) were independently correlated with RFS. MVI is an established histoprognostic factor that can only be assessed on surgical specimens. Nevertheless, SP70 can complement this limitation as a surrogate biomarker. To date, most studies have considered that continuous increase in AFP levels is a risk factor for HCC development. However, there has been no agreement on the use of AFP for surveillance and the best cut-off value for clinical practice (34–37), which may be due to a considerable number of patients with negative AFP. Whether preoperative AFP can be used to predict early recurrence of HCC is still controversial. In a large multicenter study, Farinati et al. (38) assessed the prognostic reliability of AFP and indicated that AFP correlated with overall survival in transplanted patients, patients undergoing locoregional treatments or untreated patients, but not in those undergoing resection. In addition, Calderaro et al. (39) showed that high levels of serum AFP (>300 ng/ml) were not significantly associated with the risk of early tumor recurrence. In our study, serum AFP was an independent factor for predicting early recurrence. Nevertheless, it was still negative in 43.5% of AFP-negative HCC patients at the time of recurrence. Our result showed that a high level of SP70 was associated with significantly worse RFS in the AFP-negative subgroup, indicating that SP70 can be used as a sensitive predictor for early recurrence, especially for AFP-negative patients. In addition, SP70 is involved in regulating gene expression related to migration and invasion of tumor cells. SP70 blocked could upregulate the expression of tissue inhibitor of metalloproteinase 3 (TIMP3) (40). The expression and involvement of TIMP3 in regulating the growth, migration and invasion of HCC and other tumors have been determined in several studies (41–43). As a tumor-derived antigen, SP70 induces immunosuppression in a tumor microenvironment by augmenting Tregs differentiation, potentially mediating tumor immune escape and anti-tumor immune collapse, according to our ongoing research. Based on the above evidence, we propose that SP70 may play a critical role in early metastasis of HCC and can be a sensitive indicator in detecting early recurrence after resection.

This study is the first to demonstrate the diagnostic and prognostic role of SP70 in HCC, affirming its clinical predictive value and application prospect. Undeniably, our study has some limitations. This is a single-center study, that may lead to potential bias in specimen and data collection. Another limitation is that data on AFP-L3 detection is not provided in the comparison of markers. Finally, data on the characteristics of SP70 in patients with unresectable HCC and its assessment of overall survival were not provided. The follow-up period needs to be extended in further studies.

In conclusion, SP70 is highly expressed on the membrane and in the cytoplasm of HCC cells. SP70 could be used as a valuable biomarker for HCC, and a combination of SP70 with AFP enhances diagnostic efficiency compared to individual biomarkers and a combination of AFP and PIVKA-II. Furthermore, SP70 is significantly and independently associated with RFS and could be a sensitive indicator in predicting early recurrence of HCC.

## Data availability statement

The raw data supporting the conclusions of this article will be made available by the authors, without undue reservation.

## Ethics statement

The studies involving human participants were reviewed and approved by Institutional Ethics Committee of the First Affiliated Hospital of Nanjing Medical University. The patients/participants provided their written informed consent to participate in this study.

## Author contributions

Study design, guidance in writing of the manuscript, and review of the final manuscript: S-YP. performance of experiments: HS, Y-XJ. collection of the data: JW, W-XC, C-RG, YM and JX. analysis and interpretation of data: LW and HS. statistical analysis: LW and YM. writing of the manuscript: LW. All authors contributed to the article and approved the submitted version.
